# Enchondromatosis of Both Hands With Possible Skull Bone Involvement: An Extremely Rare Condition in Adults

**DOI:** 10.7759/cureus.95785

**Published:** 2025-10-30

**Authors:** Mihaela Pertea, Malek Benamor, Alexandru - Hristo Amarandei, Stefana Avadanei-Luca, Andra - Irina Bulgaru - Iliescu, Raluca Tatar

**Affiliations:** 1 Surgery, Grigore T Popa University of Medicine and Pharmacy, Iași, ROU; 2 Department of Plastic Surgery and Reconstructive Microsurgery, Sf Spiridon Emergency County Hospital, Iași, ROU; 3 Plastic Surgery and Recontructive Microsurgery, Sf Spiridon Emergency County Hospital, Iași, ROU; 4 Surgery, Grigore T Popa University of medicine and Pharmacy, Iași, ROU; 5 Department of Plastic Reconstructive Surgery and Burns, ‘Grigore Alexandrescu’ Children’s Emergency Hospital, Bucharest, ROU; 6 Plastic Surgery, Carol Davila university of Medicine and Pharmacy, Bucharest, ROU

**Keywords:** chondrosarcoma, enchondromatosis, excision, hands, ollier disease

## Abstract

Multiple enchondromatosis (Ollier Disease) is characterized by the presence of enchondromas predominantly affecting long and flat bones, and only exceptionally involving cranial bones. The aim of this study is to highlight the extreme rarity of bilateral hand involvement in Ollier disease, occurring simultaneously with other unusual localizations - such as the scapula - and exceedingly rare ones, such as the vomer bone. Furthermore, the study emphasizes the psychological impact of this pathology and the need for appropriate psychological and psychiatric support for affected patients. A systematic literature search was performed to identify English-language articles published between January 2015 and July 2025 concerning Ollier disease (enchondromatosis) with bilateral hand involvement in adult patients. The databases searched included PubMed, Scopus, and Google Scholar. The following search terms were used in various combinations: “Ollier disease,” “enchondromatosis,” “multiple enchondromas,” “adult,” “bilateral hands,” “case report,” “case series,” and “chondrosarcoma.” Inclusion and exclusion criteria were defined, and data were systematically recorded. In addition, we report a case of Ollier disease in a young male patient presenting with multiple rare bone lesions along with numerous comorbidities. The review confirms the exceptional rarity of cases involving both hands, with only five such reports identified in the analyzed literature. No cases of malignant transformation were reported in the studies included in this review. Ollier disease is a severe, debilitating pathology with limited therapeutic options and a significant risk of malignant transformation.

## Introduction and background

Multiple enchondromatosis - Ollier disease (OD), first described in 1889 by Louis Léopold Ollier, is a rare, non-hereditary pathology that occurs sporadically [1]. It is most often diagnosed in early childhood due to the appearance of painful bone deformities, limb asymmetry, and growth disturbances. The disease has an estimated incidence of 1 in 100,000 individuals [2]. OD most commonly affects the bones of the hands, with the phalanges being most frequently involved, followed by the metacarpal bones, and less frequently the long bones such as the femur, tibia, fibula, humerus, radius, and ulna [3]. There are a few reported cases involving flat bones, such as the scapula, and extremely rare cases affecting the bones of the skull. The etiology and pathogenesis of the disease remain poorly understood. Over time, several theories have been proposed [3]. In 2006, Silve and Jüppner suggested a link between OD and somatic mutations in the parathyroid hormone receptor 1 gene (PTHR1), specifically the R150C mutation [4]. Five years later, in 2011, Amary et al. associated Ollier disease with somatic mosaic mutations in the IDH1 and IDH2 genes [5,6]. The multiple enchondromas in OD show an uneven distribution and may be unilateral or symmetrical, varying in number and at different stages of evolution [7]. Diagnosis is based on two main components: clinical examination and imaging studies [8]. Histopathological diagnosis is valuable in identifying malignant transformation (into chondrosarcoma or osteosarcoma), which occurs in 5% to 50% of cases [8]. Since 2013, the World Health Organization (WHO) has classified chondrosarcoma into three subtypes based on their degree of malignancy: low-grade, intermediate-grade, and high-grade. Grade I chondrosarcoma is an “atypical cartilaginous tumor” (ACT), recognizing it as a rarely metastasizing tumor and classifying it as an intermediate-grade neoplasm rather than a fully malignant tumor [9].

The main clinical symptoms include pain, bony edema, visible and/or palpable deformities, limb asymmetry with or without bone distortion, and the possible occurrence of pathological fractures [10]. These clinical findings are confirmed by imaging: plain radiography typically reveals radiolucent masses within the bone, centrally located, with or without cortical destruction depending on tumor size, often involving the growth plate [1,3]. Advanced imaging techniques such as MRI and CT scans are valuable for evaluating the extent of lesions and detecting potential malignant transformation. Bone scintigraphy can also be used to screen for malignant changes. Imaging is further helpful in identifying other conditions associated with OD, such as skull base gliomas, pancreatic neoplasms, and hematological disorders [10]. Histopathological analysis is essential for determining whether malignant transformation has occurred. Initially, enchondromas are benign cartilaginous tumors developing in the metaphysis of growing bones, later affecting the diaphysis and potentially infiltrating surrounding soft tissues [11]. These tumors can cause extensive bone destruction and deformation, leading to severe disability and significant emotional impact on the patient, beginning in childhood and continuing into adulthood [12]. This has profound implications for the patient’s psychological well-being. Although OD is rare and debilitating, with a high risk of malignant transformation, there is currently no specific treatment available [3]. Patients with small, asymptomatic enchondromas that do not cause significant functional impairment are typically monitored over time. Surgical treatment is reserved for complications such as limb shortening, deformities, pathological fractures, and malignant transformation [13]. Interventions may include bone lengthening procedures, deformity corrections, tumor resections, and, in some cases, limb amputations [7,10].

This paper includes a systematic review of the literature on OD affecting both hands, with or without associated skull bone lesions from the past 10 years (2015-2025). The limited number of reports included in the review highlights the rarity of multiple enchondromatosis cases involving both hands with or without associated skull bone lesions. Reported surgical interventions are generally limited to tumor excision or, in more advanced cases, amputation, usually when the lesions are large or show evidence of malignant transformation. No alternative surgical or non-surgical therapeutic approaches have been reported to date. Notably, CT imaging also confirmed the presence of an enchondroma in the bones of the skull, an extremely rare location.

## Review

Materials and methods

A systematic literature search was conducted to identify English-language articles published between January 2015 and July 2025 on OD (enchondromatosis) with bilateral hand involvement in adult patients. Databases searched included PubMed, Scopus, and Google Scholar. The following search terms were used in various combinations: “Ollier disease”, “enchondromatosis”, “multiple enchondromas”, “adult”, “bilateral hands”, “case report”, “case series”, and “chondrosarcoma”. Reference lists of retrieved articles were also manually screened to identify any additional eligible publications.

Studies were eligible for inclusion if they met all of the following criteria: published in a peer-reviewed English-language journal between 2015 and 2025, described adult patients (age >18 years) diagnosed with OD or multiple enchondromatosis, provided documentation of enchondromas in both hands (initial or progressive involvement), included information about the clinical presentation, lesion localization, and imaging findings, mentioned treatment strategy (surgical or non-surgical) and patient outcomes, reported unusual localizations of enchondromas - skull bones.

Exclusion criteria were pediatric-only cases, reviews lacking individual case-level data, non-English publications, and abstracts without full-text availability. All included studies were analyzed manually. Extracted data included: author and year, type of study (case report, case series, literature review), number and demographic profile of cases, anatomic distribution of enchondromas (hands and other bones), imaging modalities used and radiological findings, histological diagnosis and presence of malignant transformation, type of treatment received, evolution, and clinical outcome.

Studies of Maffucci syndrome (enchondromatosis with hemangiomas) were excluded. Due to the rarity and heterogeneity of cases, meta-analysis was not feasible, and only a descriptive synthesis of findings was conducted. The study was not registered.

Results

Five studies (each describing a single case) met the eligibility criteria. Data were extracted on patient demographics, lesion distribution, diagnostic imaging, treatments, occurrence of malignancy or other comorbid conditions, and clinical course.

**Figure 1:**
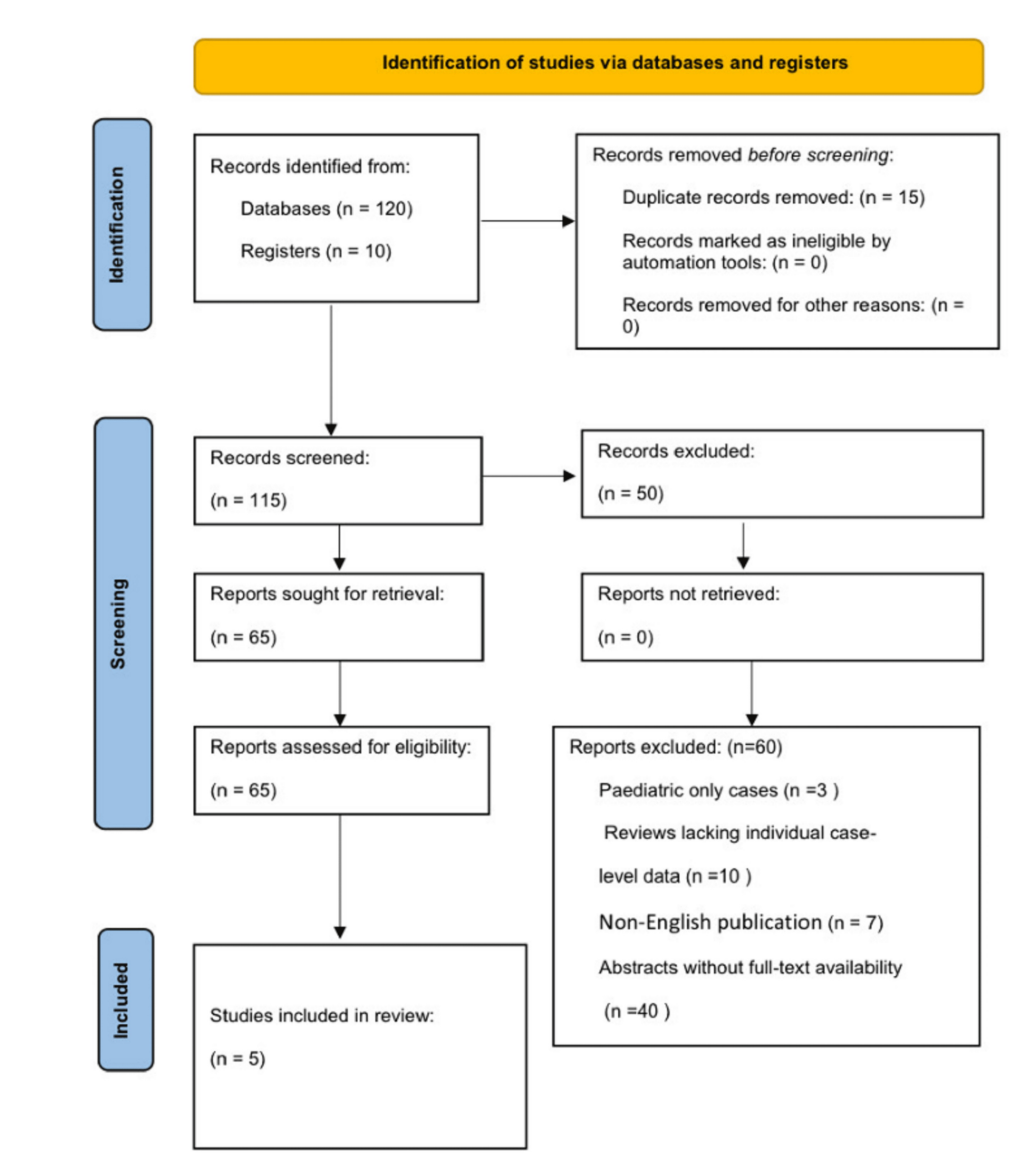
PRISMA flow diagram of literature search and study selection for the systematic review. The diagram illustrates the number of records identified, screened, excluded, and included at each stage.

An updated PRISMA flow diagram of study selection is shown in Figure 1.

All included patients had multiple enchondromas involving the hands (metacarpals and phalanges) along with other upper limb skeletal sites (Table 1).

**Table 1:** Literature reports of enchondromas in both hands with or without associated skull bone lesions between 2015 and 2025 AML: acute myeloid leukemia

Author/Year	Patient (Sex, Age)	Distribution	Imaging	Treatment(s)	Malignity (Bone Lesions)	Other Localizations/Comorbidities/Complications	Outcome/Follow-up
Sabeti et al. (2015) [12]	F, 25-year-old	Both hands (phalangeal), skull base (clival) lesion	X-rays - skull MRI - brain	Trans-sphenoidal resection of skull base enchondroma; no hand surgery	Benign	Intracranial enchondroma causing cranial nerve palsy (ptosis, vision loss)	No neurologic symptoms, no recurrence reported, no malignant transformation
Lipatov et al. (2021) [14]	M, 39-year-old	Both hands and feet have multiple long bones in legs (previous surgeries on feet)	X-rays - hand MRI - hand	Urgent surgical debridement of infected proximal phalanx, antibiotics prior to orthopedic surgeries on enchondromas of feet	Benign	Infection- osteomyelitis of the right ring finger, enchondroma (post-insect bite)	No infection, hand function preserved; enchondromas otherwise stable; no malignancy at 1-year follow-up
Fadili et al. (2023) [15]	M, 25-year-old	Both hands and left foot	X-rays - hand	Left trans-metacarpal hand amputation at age 21	Benign	New enchondromas arose in right hand and foot by age 25	No malignant transformation, significant disability from left-hand loss
Lau et al. (2024) [16]	M, 25-year-old	Both hands (P, MC) long bones (bilateral tibias, left fibula), bilateral feet, shoulder, ribs	X-rays - hand, legs, whole-body bone scan	No surgery, physical therapy; planned serial imaging (annual)	Benign	No malignancy, no systemic disease	Stable over 8-year follow-up, no new symptoms, regular monitoring for any sign of malignant change or fractures
Corvino et al. (2024) [17]	M, 38-year-old	Both hands (P)	X-rays - hand bone marrow biopsy, MRI-brain, PET, genomic testing (IDH1)	Enchondroma excisions, chemotherapy stem cell transplant for AML, craniotomy and resection for brain glioma, rehabilitative therapy	Benign	Acute myeloid leukemia (diagnosed at age 35), diffuse midline glioma of the brainstem (IDH-mutant)	Lifelong surveillance (IDH1-mutant) AML: in remission posttransplant patient under close follow-up, no malignant transformation

Study and patient characteristics

Five case reports (2015-2024) of adult OD with bilateral hand involvement were included. The patients ranged in age from 25 to 39 years (mean ~30 years) and included four males and one female. All patients had multiple enchondromas affecting both hands (metacarpals and phalanges). In several cases, the disease predominantly involved one hand initially, with later contralateral hand lesions developing. Besides the hands, enchondromas were frequently present in other skeletal sites: three patients had lesions in the feet, two had long bone involvement of the limbs, and one had pelvic/axial lesions, including the skull base. Notably, none of the five patients had developed a conventional chondrosarcoma of the appendicular skeleton at the last follow-up, despite the known malignant potential of OD. However, serious disease-related complications were reported in several cases, including malignant transformation to extra-skeletal cancers. Only one reported case documented the presence of an intracranial enchondroma that produced significant clinical symptoms and was, in fact, the primary finding leading to the diagnosis of the disease [12]. Additionally, only one case described a history of multiple prior surgical procedures performed for the excision of enchondromas [14]. In none of the reviewed cases was there any mention of psychiatric disorders emerging in the context of OD diagnosed during early childhood.

Diagnosis, Imaging, Histopathology

All cases were diagnosed based on characteristic clinical and radiographic findings of enchondromatosis, supplemented by advanced imaging or histopathology as needed. Plain radiographs of the hands demonstrated the pathognomonic appearance of multiple lucent, expansile lesions with chondroid matrix calcifications in the metacarpals and phalanges.

Sabeti et al. (2015) vividly show a plain X-ray of both hands revealing numerous enchondromas with calcifications [12]. X-rays can show bone destruction at the level of the hand bones.

In one case, whole-body bone scintigraphy was used to map the full extent of skeletal involvement, showing multifocal tracer uptake in the hands, feet, and other sites consistent with enchondromatosis. CT and MRI exams were employed selectively: to evaluate a skull base lesion causing neurologic symptoms, to characterize suspected malignant transformation, and to assess a diffuse brainstem lesion in one patient.

A CT scan can also provide more eloquent images of bone destruction. In all patients, the diagnosis of OD was ultimately confirmed either by classical radiographic features or by histological examination of excised tissue. There are histopathological features of malignancy that indicate malignancy of the endochondroma.

It is noteworthy that in none of the cases included in the present review was malignant transformation identified either intraoperatively or during postoperative follow-up. The presence of enchondromas involving cranial bones is extremely rare and has been reported only in a case described by Sabeti, in which a trans-sphenoidal resection was required [12].

Treatments

Management strategies varied depending on symptomatology, anatomical location of the bone lesions, associated comorbidities, and emerging complications. Four out of five patients required surgical intervention at some point, underscoring the frequent need for operative management in this cohort. One patient, who had a severely progressive disease course, underwent a trans-metacarpal amputation of the left hand at age 21 (with preservation of the fifth finger) due to extensive enchondromatous destruction and complete loss of hand function. Another patient underwent multiple curettage procedures or resections of enchondromas affecting the fingers of both hands during childhood and adolescence, as well as orthopedic surgeries for lesions located in other skeletal sites. In the case involving an enchondroma at the base of the skull, neurosurgical resection was performed via a trans-sphenoidal approach to relieve brainstem compression and visual symptoms [12]. One patient developed acute osteomyelitis in an enchondroma of the right ring finger and was successfully treated with surgical debridement, specifically, osteonecrotomy of the affected phalanx and excision of surrounding enchondromatous tissue, alongside targeted antibiotic therapy [14]. The only patient who did not undergo surgery was a 25-year-old woman with relatively mild disease limited primarily to the hands; she was managed conservatively with serial imaging and has not required surgical intervention to date [16]. All patients were advised to undergo regular surveillance imaging (typically annual radiographs or periodic MRI) to monitor for lesion progression or malignant transformation [12-17].

Comorbidities

In none of the reviewed cases was malignant transformation into chondrosarcoma observed. However, it is important to underscore the possibility of cranial bone involvement by enchondromas, which may present with neurological symptoms that serve as clinical clues. Multiple enchondromatosis may also be associated with other pathologies. In the present review cohort, Corvino et al. (2024) described a 38-year-old man with a long-standing history of OD (involving both hands) who developed acute myeloid leukemia (AML) at the age of 35, followed one year later by a diffuse intrinsic brainstem glioma [17]. Notably, the IDH1 R132H mutation was detected in the patient’s enchondroma tissue, leukemic cells, and glioma, suggesting a shared oncogenic pathway. The patient was treated with systemic chemotherapy and underwent stem cell transplantation for AML (achieving remission), followed by a craniotomy and gross total resection of the high-grade glioma. This unique case illustrates that OD may, in rare instances, be associated with other neoplasms arising from a common IDH1-mutant pathogenesis [17].

Discussion

Enchondromatosis represents a group of rare skeletal disorders characterized by multiple enchondromas, benign cartilaginous tumors that originate from remnants of growth plate cartilage and persist within the medullary cavity [3, 18]. OD and Maffucci syndrome are non-hereditary conditions involving multiple enchondromas, most commonly affecting the long bones and small bones of the hands. A major concern in these disorders is the increased risk of malignant transformation into secondary chondrosarcoma. Although enchondromatosis may be detectable at birth, clinical manifestations typically do not appear until around the age of five [19].

Multiple enchondromatosis is classified into six subtypes: OD, Maffucci syndrome, metaphyseal chondromatosis, metatarsal chondromatosis, interstitial spinal chondroplasia, and interstitial spinal chondromatosis [11, 20]. Based on radiological features, the affected bones, and the manner of disease acquisition, Spranger (1978) classified the disease into Spranger Type I and Type II, the latter corresponding to Maffucci syndrome [21]. The disease is characterized by asymmetrical distribution of bony tumors, primarily involving the tubular bones. When OD affects the femur, tibia, or humerus, the most common site (50%) is the metaphysis; in 33% of cases, the diaphysis is involved, and in the remaining 17%, the epiphysis. The small bones of the hand are more frequently affected (43.7%) compared to those of the foot (18.5%) [22]. Among these, the phalanges are more often involved than the metacarpals, which are frequently asymptomatic. Intracranial or cranial bone involvement by enchondromas has been reported in a few isolated cases [12, 23]. The first reported case of an intracranial chondrosarcoma in a patient with OD was published by Reuter and Weber in 1981 [24]. Up to 2018, seven intracranial enchondromas had been reported in patients aged 18 to 36 years (mean age 27.8), all of whom were female [25]. These lesions were located in the sphenoid bone, sella turcica, suprasellar region, cavernous sinus, clivus, and petrous apex. Another rare localization in OD is the scapula, which has been mentioned only exceptionally [26].

Diagnosis is based on characteristic clinical features and imaging findings [27]. Cranial bone involvement may cause neurological symptoms or may remain clinically silent, making diagnosis more challenging or delayed. Given that the average age of diagnosis for OD is around 7 years, it is uncommon for the diagnosis to be made following a minor trauma, as typically occurs in solitary enchondromas when a fracture reveals a pathological bone lesion [22]. In most reports, the definitive diagnosis was established by histopathological examination. Histology is not primarily used to confirm the disease itself but is crucial for detecting signs of malignant transformation, such as nuclear polymorphism, hyperchromaticity, and abnormal mitotic figures [18].

The largest case series on chondrosarcomas in patients with OD and Maffucci syndrome was published by Vedagall et al. in 2011, including 144 patients with OD and 17 with Maffucci syndrome [28]. Nevertheless, Prokopchuk (2016) reported that the risk of malignant transformation is higher in Maffucci syndrome (52-57.1%) than in OD (20-45.8%) [29]. Chondrosarcoma has been most frequently reported in the long bones of patients with OD or Maffucci syndrome [22].

None of the cases included in the current review demonstrated malignant transformation.

Imaging plays a central role in diagnosis. Conventional radiographs are complemented by CT and/or MRI. When these modalities are not available, bone scintigraphy may be considered. CT was performed instead of MRI due to financial limitations. MRI is the preferred method for long-term surveillance and should ideally be performed annually [22].

Surgical treatment generally consists of tumor excision, with or without filling of the resulting bone defect using bone substitutes and, in some cases, internal fixation. When tumors are large and accompanied by severe deformities with no reconstructive options, amputation may be required [7, 30]. No adjuvant therapies have been reported. Associated comorbidities should be managed as part of the treatment plan. To date, no curative or disease-modifying therapies have been identified that could slow or halt disease progression [30].

The presence of multiple enchondromas, their potential to appear in any anatomical region, the progressive deformities they cause, the occurrence of fractures in weakened bone, and the resulting disabilities, combined with early onset in childhood, associated comorbidities, frequent hospitalizations, and repeated surgeries, place these patients at high risk for psychiatric disorders. For this reason, psychiatric support in OD should be considered essential.

Finally, publication bias cannot be ruled out, as the studies are small in number and the existing cases could have remained unpublished. Since no formal assessment of publication bias (e.g. funnel plot or Egger test) could be performed due to the lack of restricted data, despite these limitations, the analysis provides a comprehensive synthesis of what Ollier's disease in adults means, the possible presence of bone tumors (enchondromas) at known anatomical levels such as the hand but also the presence of both hands, scapula, long bones but also at the level of the skull bones. Future systematic studies will be needed to analyze other aspects of this disease and also the presence of enchondromas in other bones, which have not been reported so far.

## Conclusions

Multiple enchondromatosis (Ollier disease), characterized by the presence of benign cartilaginous tumors (enchondromas), is typically diagnosed during the first years of life. The distribution of enchondromas is diffuse and asymmetric, with bilateral hand involvement being exceedingly rare. These tumors primarily affect long and flat bones but can also appear in other regions, including some previously extremely rare sites. Clinical and imaging examinations establish the diagnosis, while histopathological analysis is essential for detecting potential malignant transformation. The deforming bone involvement, frequent pathological fractures, and growth disturbances render the disease profoundly debilitating. There remains an unmet need for effective medical therapies aimed at modulating disease progression, preventing complications, and reducing the risk of malignant transformation. Surgical treatment is limited to tumor excision, fracture management, and amputations. The management of these patients must also include psychological and psychiatric support, as they are often young individuals with recurrent hospitalizations, significant functional and aesthetic disabilities, and, in many cases, multiple associated comorbidities. Considering these factors, Ollier disease follows a progressive course with an unpredictable prognosis.
